# Complete mitochondrial genomes of three *Saccharomyces cerevisiae* flor strains

**DOI:** 10.1080/23802359.2017.1407699

**Published:** 2017-11-25

**Authors:** Andrey V. Mardanov, Alexey V. Beletsky, Mikhail A. Eldarov, Tatiana N. Tanashchuk, Svetlana A. Kishkovskaya, Nikolai V. Ravin

**Affiliations:** a Institute of Bioengineering, Research Center of Biotechnology of the Russian Academy of Sciences, Moscow, Russia;; b Research Institute of Viticulture and Winemaking “Magarach” of the Russian Academy of Sciences, Yalta, Russia

**Keywords:** Flor yeast, mitogenome, *Saccharomyces cerevisiae*

## Abstract

The complete mitochondrial genome sequences of three *Saccharomyces cerevisiae* flor strains used for the production of sherry-like wines in Russia were sequenced. The phylogenetic analysis showed that despite their origin from different georgaphic regions, these strains formed a single cluster, also including the wine yeast strain YJM 270.

*Saccharomyces cerevisie* are widely used in the food industry for fermentation of various substrates. *S. cerevisiae* strains used in the production of sherry-like wines are able to form a biofilm (‘flor’) on the surface of the wine in course of its maturation (Alexandre 2013). Flor yeasts can oxidize ethanol with the formation of aldehydes, acetales and other compounds responsible for peculiar flavour of sherry. Comparative genomic studies of *S. cerevisiae* strains used for the production of wines, beer, cider and other spirits uncovered their evolutionary history and genetic properties (Borneman et al. 2008; Wolters et al. 2015; Coi et al. 2017). These studies mostly involved flor yeast strains from Spain and France where sherry-like wines were traditionally produced.

In this work, we determined complete mitochondrial genome sequences of three *S. cerevisiae* flor strains from the The Magarach Collection of the Microorganisms for Winemaking that are used for production of sherry-like wines in Russia. Two strains, I-566 and I-30, were isolated from industrial yeast flors in Armenua and Crimea, respectively. The third strain was originally obtained from the Spain and subsequently improved by selection (Kishkovskaia et al. 2017).

For each strain, a True seq DNA library was prepared and sequenced using Illumina HiSeq2500 genome sequencer (250-nt reads). Sequence primers were removed using Cutadapt (Martin 2011) and low-quality read regions were trimmed using Sickle (https://github.com/najoshi/sickle). Illumina reads were *de novo* assembled using SPAdes 3.7.1 (Bankevich et al. 2012). Contigs representing complete mitochondrial genomes (mtDNAs) were identified by BLASTN searches against *S. cerevisiae* S288C mtDNA sequence.

The mtDNAs of strains I-30, I-329 and I-566 are 87,571, 80,204 and 80,886 bp long, respectively. They were annotated using MFannot (Beck and Lang 2010) following a manual curation. Like in other *S. cerevisiae* (Foury et al. 1998), all three mtDNAs contain a common set of protein-coding genes that includes ATPase subunits (*atp6, atp8, atp9*), three subunits of cytochrome C oxidase (*cox1, cox2, cox3*), apocytochrome b (*cob*) and ribosome-associated protein (*VAR1/rps3*). The order of these genes in all three mtDNAs is identical. In addition, 6, 4 and 3 open reading frames coding for LAGLIDADG endonucleases (pfam00961 domain) were predicted in mtDNAs of strains I-30, I-329 and I-566, respectively. All three genomes contain genes for large and small subunits of rRNA, 24 tRNA genes and the *rnpB* gene.

To reveal the evolutionary history of the three analysed flor yeast strains, we made phylogenetic analysis based on concatenated sequences of eight mitochondrial genes (Figure 1). In addition to I-30, I-329 and I-566, seven other *S. cerevisiae* strains were included in the analysis: YJM975 (clinical), YJM1417 (cider), YJM189 (distillery), YJM1415 (wine), YJM1433 (wine), YJM1574 (wine) and YJM270 (wine). Phylogenetic analysis showed that the three flor yeast strains from the Magarach collection, despite their origin from georgaphically remote regions, formed a single cluster. Interestingly, this cluster also included wine strain YJM 270 (CBS 2807) from Slovenia. Although CBS 2807 was not described as a flor yeast strain, it is alcohol resistant and produces up to 17–18% of ethanol (http://www.cabri.org/HyperCat/yeast/all36889.htm), which is comparable with the concentration of ethanol in sherry.

**Figure 1.**
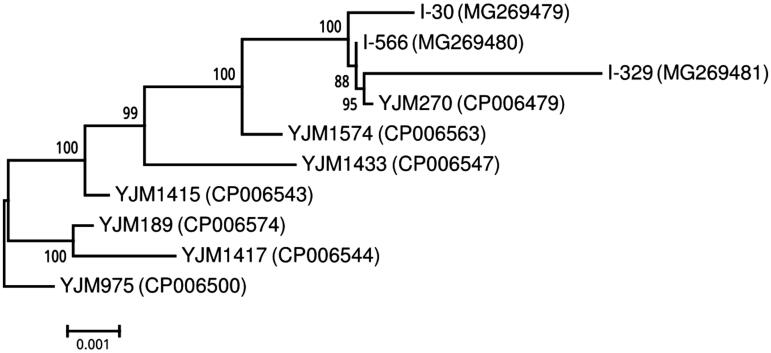
Phylogenetic tree of strains I30, I329 and I566, and seven other *S. cerevisiae* strains. The tree is based on concatenated nucleotide sequences of genes *cox1*, *atp8*, *atp6*, *cob*, *atp9*, *rps3* (var1), *cox2* and *cox3*. Alignment was performed using Muscle v 3.8.31. The tree was constructed using the maximum likelihood method in PhyML v. 3.2.20160531. The branch support values, displayed at each node, were calculated using the approximate Bayes method. GenBank accession numbers are shown in parenthesis after the strain names.
